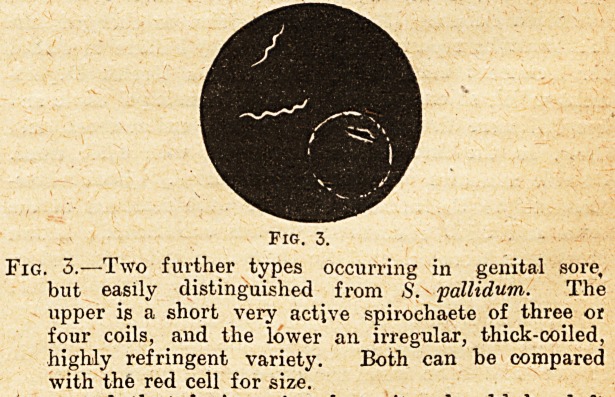# Early Diagnosis of Syphilis

**Published:** 1919-03-01

**Authors:** 


### EARLY DIAGNOSIS OF SYPHILIS.

### The Means in Practitioners' Hands.

One cannot over-estimate the importance of a subject affording the earliest means of diagnosis in syphilis, and this at a period when clinically it is otherwise almost impossible to arrive at a definite decision. It is known that treatment in the very early primary stage is relatively simple and assured, whereas after generalisation of the infection and the development of a positive Wassermann reaction it is prolonged, uncertain and a matter of months. For thoroughness one must suspect every localised lesion, however small or atypical, as being of possible spirochaetal origin if on the organs of generation, and, seeing that the most reliable methods of detection are of the simplest nature, it should become a routine practice to examine every such eruption for the organism. There is no excuse for the omission by any organised institution, and even for the practitioner/ the outlay on apparatus is so small compared with the value of the result that the means may well form part of his equipment. The following details are largely drawn from the report of the Medical Research Committee, but in their original form they carry differentiation to a stage of such completeness that we may omit some of the finer points and yet outline a thoroughly efficient scheme for the solitary worker.

### Collection of Material.

It is necessary to examine *uncontaminated fluid freshly expressed from the lesion.* Also care must be taken that previous local antiseptics do not gain access to the specimen, and it must be borne in mind that salvarsan or its substitutes have an extremely rapid action on the spirochaetes, so that microscopical diagnosis must be made prior to arsenical treatment.

(*a*) Superficial Lesions.—Sore is thoroughly cleansed and dried, squeezed until drops of serum are expressed on the surface, and this drawn by capillary attraction into small pipette, the end of which has been applied to the fluid. Traces of blood must be avoided, as the corpuscles spoil the microscopic field if present in any quantity.

(*b*) Skin Lesions.—Serum may be obtained by scarifying or the use of a blister and the extraction of fluid from the raw area exposed.

(*c*) Lymph Glands.—About 5 minims of salt solution (0.85 per cent.) are injected into the centre of the gland through a stout hypodermic needle, the gland massaged, and some of the fluid drawn back into the syringe, and then transferred to a slide.

### Demonstration of Spirochaetes.

(1) *Dark-ground Illumination.*—This is undoubtedly the ideal method. A thin slide is used, and a fine cover slip laid gently upon the specimen. Any good microscope suited to bacteriological work can be adopted by fitting the special type of condenser, and by using a strong, constant beam of light. The apparatus can be readily obtained from manufacturers, and as complete details of action and mechanism are provided with it, it is unnecessary to describe more than the principle involved. In the field of such a microscope an object becomes self-luminous, so that no previous staining or fixing is necessary, and any suitable material may be examined in the living state. The most satisfactory illuminant is that made by the Ediswan Company, and known as the "Pointolite" lamp. The conditions necessary to achieve self-luminosity are that the structure or organism should have a refractive index differing substantially from the medium in which it lies, and that it should be illuminated in such a manner that only the light refracted or reflected by the object can reach the eye of the observer. Under these conditions greater visibility is secured, and the object is seen as a bright image on a dark ground. The accompanying small diagrams roughly illustrate the essential points by which *S. pallidum* may, on the dark ground field, be distinguished from other spirochaetes occurring on genital sores.

Fig. 1.

Fig. 2.

Fig. 1.—*S. pallidum*, with very fine coils, for the most part clear-cut spaces, and, as seen by comparison with the accompanying red cell, about seven coils to the diameter of the cell. Colour blue-white and never dazzling.

Fig. 2.—Spirochaete from genital sore. Comparison with fig. 1 shows it to have thicker coils, and five of these to the diameter of a red cell. Also it is more refringent and often very brilliant in the field.

The above spirochaetes are compared as the only two likely to cause confusion in diagnosis from genital sores. Types almost indistinguishable from *S. pallidum* occur inside the mouth, but it is suggested that lesions in that site should be left to those who specialise in the subject.

Fig. 3.

Fig. 3.—Two further types occurring in genital sore, but easily distinguished from *S. pallidum.* The upper is a short very active spirochaete of three or four coils, and the lower an irregular, thick-coiled, highly refringent variety. Both can be compared with the red cell for size.

**Early Diagnosis of Syphilis—(*continued*).**

The microscopic method is not at all complicated once the simple mechanism of obtaining correct position of condenser and light is mastered. The differentiation of the organisms from the sores is relatively simple once the eye is accustomed to their appearance, rate and type of movement, and it is by no means a method which must be left to the bacteriologist alone.

(2) *Staining methods.*—In practice, the preparation of specimens by staining methods takes longer; many organisms are not stained and the observer loses the advantage of seeing a living field. There are a number of available procedures, all readily found in the literature, but it is the experience of the Committee that the Romanowsky staining in one or other of its modifications affords the best results, more particularly Giemsa's long method, although Leishman's and Homer Wright's are not far behind. In all *S. pallidum* is coloured rose pink, while other spirochaetes are blue in colour, but it is well known that the organism has a notable lack of affinity for most stains, and thus only an occasional individual may be made out in a specimen actually full of spirochaetes, giving a very erroneous idea of their frequency. Indian ink and Congo red methods are considered more unreliable, since, although all spirochaetes may be very easily demonstrated, it is very hard to decide on *S. pallidum* when there is so much possible distortion according to the thickness of the film.

Conclusion.

The point we would urge is the enormous saving of time and treatment by taking the earliest possible chance to recognise the disease and the fact that, although complicated when known by description alone, the dark ground method is relatively simple in practice and at the same time the most reliable. A short while ago many venereal centres made a practice of demonstrating these points to any practitioners who cared to co-operate with the Local Government Board schemes, and these advantages are still available. Centralisation of treatment is excellent, but the routine sending of patients to large clinics must inevitably waste some time, and this is a disease where, in its early stages, every hour is of importance. If the majority of practitioners could form a rapid diagnosis and give, when required, early treatment it would often save many weeks for the patient and the clinics from considerable expense.

## Figures and Tables

**Fig. 1. Fig. 2. f1:**
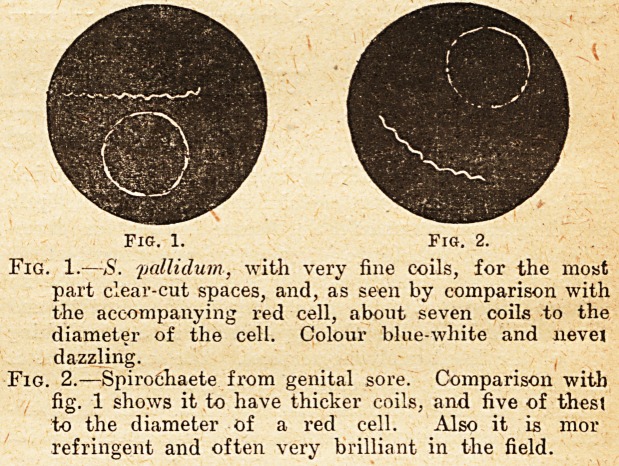


**Fig. 3. f2:**